# Unravelling the allosteric binding mode of αD-VxXXB at nicotinic acetylcholine receptors

**DOI:** 10.3389/fphar.2023.1170514

**Published:** 2023-04-13

**Authors:** Thao NT Ho, Nikita Abraham, Richard J. Lewis

**Affiliations:** Centre for Pain Research, Institute for Molecular Bioscience, The University of Queensland, St Lucia, QLD, Australia

**Keywords:** α-conotoxin, α7 nAChR, pharmacology, AChBP, allosteric, granulin fold

## Abstract

αD-conotoxins are 11 kDa homodimers that potently inhibit nicotinic acetylcholine receptors (nAChRs) through a non-competitive (allosteric) mechanism. In this study, we describe the allosteric binding mode of the granulin-like C-terminal (CTD) of VxXXB bound to *Lymnea stagnalis* acetylcholine binding protein (*Ls*-AChBP), a soluble homologue of the extracellular ligand-binding domain of nAChRs. This co-crystal complex revealed a novel allosteric binding site for nAChR antagonists outside the C-loop that caps the orthosteric site defined by the nAChR agonist nicotine and the antagonist epibatidine. Mutational and docking studies on *Ls*-AChBP supported a two-site binding mode for full-length VxXXB, with the first CTD binding site located outside the C-loop as seen in the co-crystal complex, with a second CTD binding site located near the N-terminal end of the adjacent subunit of AChBP. These results provide new structural insight into a novel allosteric mechanism of nAChR inhibition and define the cooperative binding mode of the N-terminal domain linked granulin core domains of αD-conotoxins.

## Introduction

Nicotinic acetylcholine receptors (nAChRs) are prototypical members of the ligand gated ion channels found throughout the central and peripheral nervous systems. nAChR modulation has therapeutic potential due to their association with the progression of CNS disorders including Alzheimer’s and Parkinson’s disease and schizophrenia (Dajas-Bailador and Wonnacott, 2004; Gotti and Clementi, 2004; Dineley et al., 2015). Neuronal nAChRs are assembled as α7, α8, and α9 homopentamers, or as heteropentamers comprising α2–α6 in complex with α2–α4, α7 in complex with α2 subunits, or α9 in complex with α10 subunits. The orthosteric binding pocket is located at the extracellular interface of the principal (+) and complementary (−) faces of the nAChR (Hogg and Bertrand, 2004; Jensen et al., 2005). In heteromeric nAChRs, the principal face comprises one α subunit, while the complementary face is contributed by a non-α subunit, except for α9α10 nAChRs.

Agonist binding at the orthosteric sites activates nAChRs by stabilizing the open state of the receptor with a closed C-loop conformation on the principal face. Competitive antagonists compete with orthosteric agonists for binding to this site, resulting in C-loop opening and stabilisation of the resting state (Unwin, 2005). In contrast, allosteric modulators bind to sites distinct from the binding site for orthosteric agonists to either positively allosterically modulate (PAMs) and negatively allosterically modulate (NAMs) nAChRs. nAChR PAMs either potentiate peak agonist responses (type I PAMs) or prolong channel open times (type II PAMs), while NAMs allosterically reduce agonist affinity (Hurst et al., 2005). Allosteric modulators have been identified to bind either from beneath the outermost helix of the extracellular domain, the subunit interface of the extracellular domain, the vestibule pocket opposite the agonist binding site, or the transmembrane domain (Spurny et al., 2015; Delbart et al., 2018). The sequences of these novel modulatory binding sites typically vary across nAChR subtypes, offering potential for the development of novel, subtype-selective allosteric modulators with therapeutic potential.

**FIGURE 1:**
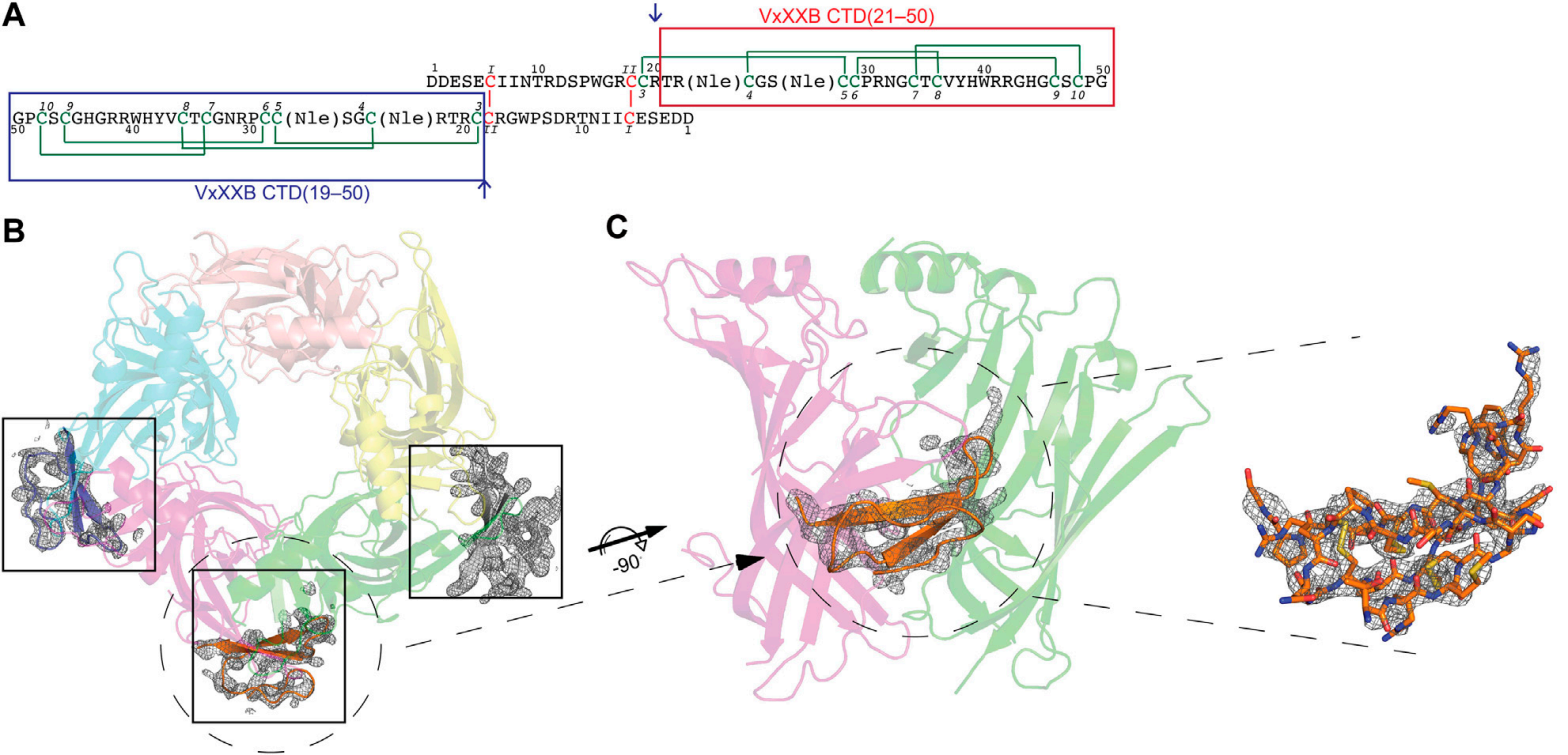
**(A)** Sequence of synthetic VxXXB with VxXXB-C(21–50) and VxXXB-C(19–50) highlighted in red and blue boxes respectively. αD-conotoxins are a symmetrical homodimer proteins (Loughnan et al., 2006) comprising two C-terminal domains (CTDs) coupled through two interchain disulfide bonds between the N-terminal domains (NTDs) where the dimerization occurs through Cys6 (Cys^I^) of one NTD chain and Cys18 (Cys^II^) of the other chain. The co-crystal structure of VxXXB-C(21–50)/*Ls*-AChBP complex viewed from top **(B)** and side **(C)**. Well-defined electron density for VxXXB-C(21–50) was seen in only three of the five potential allosteric binding sites. Two Fo-Fc map for VxXXB-C(21–50) is contoured at 1.0 Å.

Conotoxins are small disulfide-rich peptides extracted from the venom of predatory marine cone snails of the genus *Conus*. Conotoxins selectively targeting the nAChRs belong to the A superfamily, including α-, ψ-, αB, αD-, αC, and αS-conotoxins (Lewis and Garcia, 2003; Armishaw and Alewood, 2005; Lewis et al., 2012; Abraham and Lewis, 2018). α-Conotoxins are well-characterised, subtype selective competitive antagonists that plug the orthosteric site under loop C of nAChRs. In contrast, ψ- and αD-conotoxins inhibit nAChR non-competitively (allosterically) at yet to be defined binding sites (Loughnan et al., 2006). VxXXA, VxXXB, and VxXXC from the venom of *Conus vexillum* were the first D superfamily α-conotoxins characterized (Loughnan et al., 2006). αD-conotoxins are 11 kDa symmetrical homodimer proteins (Loughnan et al., 2006) comprising two C-terminal domains (CTDs or C) coupled through two interchain disulfide bonds formed between the N-terminal domains (NTDs) (Xu et al., 2015) (Figure 1A). The CTD has three disulfide bonds connected in an ICK configuration (Cys^I^–Cys^IV^, Cys^II^–Cys^V^, and Cys^III^–Cys^VI^) found in many venom peptides (Norton and Pallaghy, 1998; Xu et al., 2015), with the CTD and NTD held stable relative to each other *via* an additional disulfide bond Cys19–Cys28 (Figure 1A).

Previously, we successfully synthesised the NTD, CTD and full-length synthetic VxXXB using the α-ketoacidhydroxylamine (KAHA) ligation strategy (Ho et al., 2022). To characterize the structural basis for allosteric inhibition by αD-conotoxins, we obtained the co-crystal structure of the C-terminal domain VxXXB-C (21–50) (Figure 1) with *Lymnea stagnalis* acetylcholine binding protein (*Ls*-AChBP). AChBPs are naturally occurring soluble protein homologues of the nAChR that show remarkable structural homology, especially around the orthosteric ligand recognition site formed by aromatic side chain residues found in nAChRs (Brejc et al., 2001; Smit et al., 2001). The co-crystal structure VxXXB-C(21–50) with *Ls*-AChBP revealed for the first time the allosteric binding site of αD-conotoxins, with a separate binding site identified for the second CTD confirmed from docking-guided mutational studies on *Ls*-AChBP. Consequently, these αD-conotoxin binding determinants reveal a new allosteric site at human α7 nAChRs amenable to the rational design of novel allosteric inhibitors selective for specific nAChRs subtypes.

## Materials and methods

### AChBPs protein expression and purification

The over-expression of *Ls*-AChBP was performed as described by Abraham et al. (2016) ^21^. Ubiquitin (Ub)-tagged AChBPs were used for radioligand binding assay and de-tagged *Ls*-AChBP was used for crystallization. Briefly, Ub-tagged *Ls*-AChBPs were purified using immobilized metal affinity chromatography and the Ub tag removed by deubiquitin enzyme (produced in-house). Further purification of de-tagged *Ls*-AChBP was performed by size exclusion chromatography to assess homogeneity and oligomerization state on a calibrated analytical HiLoad 16/600 column and (GE Health Care) using AKTA FPLC system (GE Healthcare). The fractions containing the proteins were pooled and concentrated to the desired concentration using an Amicon centrifuge filter (30-kDa cut-off, Millipore).

### Mutagenesis

*L*s-AChBP mutations were introduced in the pHUE background using the QuikChange Lightning Site-directed mutagenesis kit (Agilent). Primers with the desired mutations were purchased from Sigma-Aldrich. The mutated DNA was transformed into Top 10 *E. coli* competent cells (One Shot, Thermo Fisher Scientific) and isolated *via* MiniPrep Kit (QIAGEN, Valencia, CA, United States). Successful mutations of *Ls*-AChBP were confirmed by Sanger sequencing performed at Australian Genome Research Facility (AGRF). The mutated proteins were expressed and purified as above.

### Binding assays

The ability of VxXXB variants to displace the binding of [^3^H]-epibatidine to the recombinantly expressed *Ls*-AChBP was determined in competitive radioligand binding assays (Abraham et al., 2016). Briefly, [^3^H]-epibatidine (1 nM final concentration) and increasing concentrations of test ligand in a final volume of 100 µL were incubated in 96-well plates (Flexible PET Microplate, Perkin Elmer) precoated with 1 ng/μL of *Ls*-AChBP per well in binding buffer (phosphate buffered saline with 0.05% bovine serum albumin). The mixture was then removed and 100 µL of scintillant (Optiphase Supermix, Perkin Elmer) was added to each well. Bound radioactivity was measured with a Wallac 1450 MicroBeta liquid scintillation counter (Perkin Elmer). Potency estimates of purified native VxXXB isolated from *Conus vexillum* venom (Loughnan et al., 2006; Inserra et al., 2013), which was available in limited quantities, was determined from the displacement of [^3^H]-epibatidine from *Ls*-AChBP by 10 nM and 50 nM native VxXXB, with the curve top fixed at 100% and the bottom and slope fixed to the values obtained for synthetic VxXXB.

### Data analysis

Radioligand binding data were analysed by non-linear, least squares one-site competition fits in GraphPad Prism 9.0 (GraphPad Software Inc., San Diego, CA, United States). Experiments were performed in triplicate in three independent experiments, with IC_50_ values reported as means ± S.E.M. Comparisons of the IC_50_ values of VxXXB-CTD variants at *Ls*-AChBP mutants with wildtype *Ls*-AChBP were carried out by pairwise comparison using an extra sum-of-squares F test with *p* < 0.05 in GraphPad Prism 9.0.

### Crystallization and data collection

Based on previous experimental determinations (Abraham et al., 2017; Ho et al., 2021a; Ho et al., 2021b), purified de-tagged *Ls*-AChBP and synthesized VxXXB-C(21–50) were mixed at a molar ratio of 1:2 at 4°C for 1 h before setting up crystallization trials. Crystals were successfully grown at room temperature using the hanging drop method by mixing protein and reservoir solution composed of 0.91 M lithium chloride, 16% PEG6000 and 0.1 M MES monohydrate pH 6.4 at a ratio 1:1 v/v. The crystals were cryo-protected with glycerol added to the mother liquor to a final concentration of 20% (v/v) glycerol before flash-freezing in liquid nitrogen.

### Structure determination and refinement

Diffraction data were collected at the MX2 beam line of Australian Synchrotron, Melbourne. Diffraction data were indexed, integrated *via* XDS and Molfsm and scaled *via* AIMLESS (Collaborative Computational Project, Number 4, 1994; Battye et al., 2011). The structure was solved by molecular replacement using the PHASER (McCoy et al., 2007) crystallographic software with LsIA/*Ls*-AChBP (PDB 2C9T) as search model. Refinement against experimental data was done using Phenix. refine and COOT until clear electron densities for VxXXB-C(21–50) were visible (Emsley and Cowtan, 2004; Afonine et al., 2012). NCS restraints and TLS restrains were then applied and the final structures validated with MOLPROBITY and PDB Validation (Chen et al., 2010). To simplify the building of unnatural amino acids into electron density, Met was built in place of Nle7 in VxXXB-VxXXB-C(21–50).

### Homology modelling at α7 nAChRs and α3β4 nAChRs

Homology modelling was performed using the project mode of the SWISSMODEL online server (Guex et al., 2009). Briefly, homology models were generated by aligning the ligand binding domains of the nAChR with the crystal structure of VxXXB-C(21–50) bound to *Ls*-AChBP. Similarly, homology models of native and synthetic VxXXB were generated by aligning their sequence with the crystal structure of αD-GeXXA. DeepView (Swiss-PdbViewer) was used to manually align and adjust the sequences where the conservation of structural features with functional roles was verified to ensure the correct alignment. This model was further optimized using the “project mode” of Deep View??? Finally, the resulting models were energy minimized using the GROSMACS force field in Deepview, validated *via* MOLPROBITY, and final models analyzed in PyMol (Guex et al., 2009).

### Docking of native and synthetic VxXXB at human α7 nAChRs

All docking runs were performed using the HADDOCK (High Ambiguity Driven biomolecular DOCKing) webserver (v. 2.4) (de Vries et al., 2010). To guide the docking, experimental data were translated into a collection of ambiguous interaction restraints (AIRs) on specific residues between ligands and nAChRs, including “active” and “passive” residues. Residues experimentally shown to be associated with binding were selected as “active” residues, while the corresponding set of passive residues was selected automatically by HADDOCK. AIRs were used to ensure each specified active residue was in close proximity to one or more of the active/passive residues on the partner molecule. Docking results were evaluated by MolProbity (Chen et al., 2010).

## Results

### Co-crystal structure of VxXXB-C(21–50) in complex with *Ls*-AChBP

The crystal structure of *Ls*-AChBP in complex with VxXXB-C (21–50) was solved at 2.6 Å by molecular replacement and refined to an R_free_ value of 0.29. The crystals of the protein-peptide complex belong to the P_21 21 21_ space group with cell dimensions of a = 68.91 Å, b = 119.57 Å and c = 150.73 Å. The F-loops of *Ls*-AChBP could not be constructed due to the lack of clear electron density, indicating a greater flexibility of these parts of the protein (Supplementary Table S1).

The crystal structure of *Ls*-AChBP in complex with VxXXB-C(21–50) contains one pentamer in the asymmetric unit, with the electron density of VxXXB-C(21–50) detectable adjacent to four of the five orthosteric binding sites (Figure 1B). In the four occupied orthosteric binding sites, loop-C of *Ls*-AChBP moves outward (10.23–10.90 Å based on the measurement between Cys187 C_α_ atom in the complex with HEPES/*Ls*-AChBP structure), which is a similar backbone orientation to previously characterized co-crystal structures of α-conotoxins ^14–17^. However, only three binding interfaces generated clear ligand electron density, while the fourth binding interface showed reduced electron density. The unoccupied binding site was likely to be directly affected by the adjacent crystal mate in close proximity, in contrast to the three clearly occupied binding interfaces which show significant separation with adjacent crystal mate Supplementary Figure S1. On the other hand, the binding interface with ambiguous ligand density showed a weaker influence of crystal packing on VxXXB-C(21–50) binding (Supplementary Figure S1D).

### The VxXXB-C(21–50)/*Ls*-AChBP complex

#### The structure of VxXXB-C(21–50)

**FIGURE 2:**
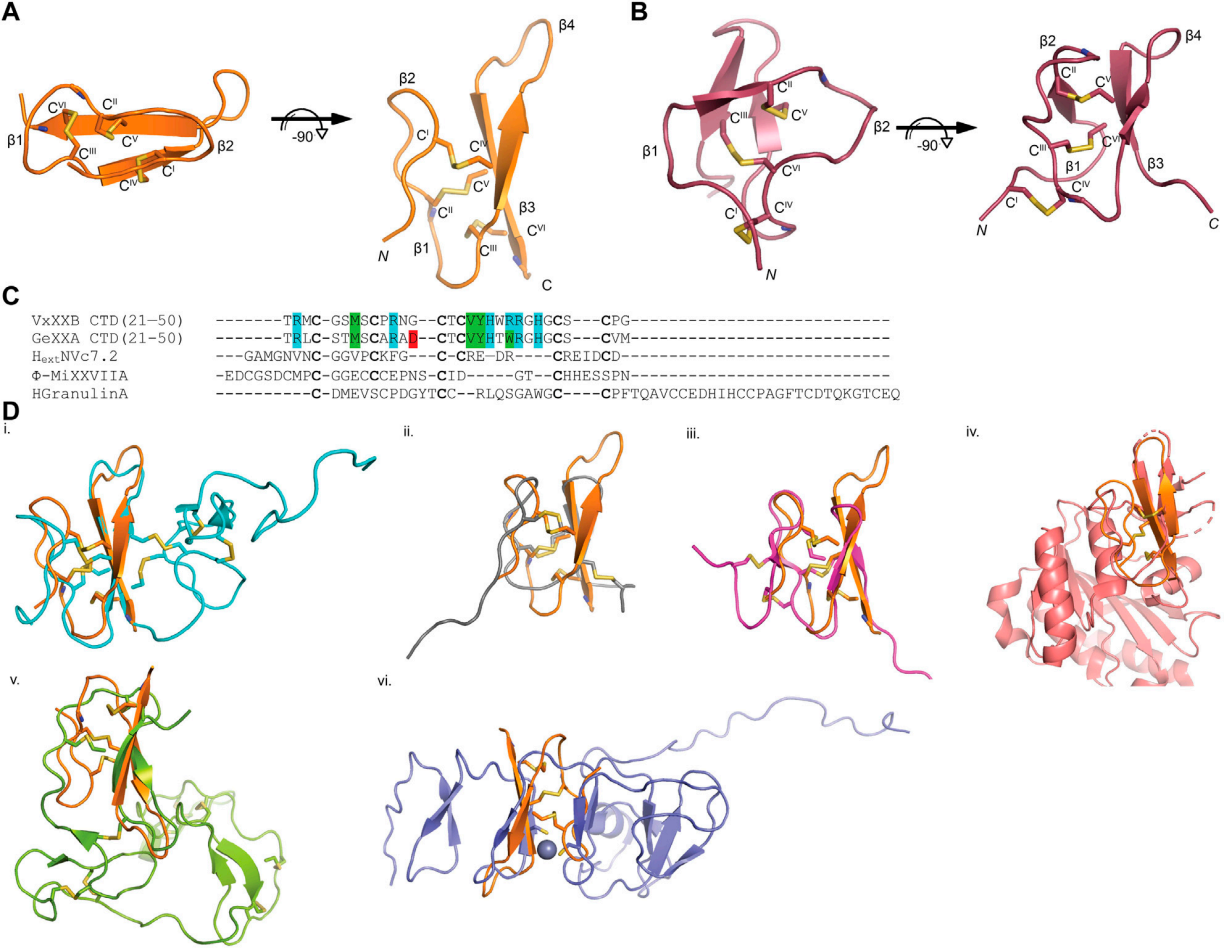
VxXXB-C(21–50) adopts a mini-granulin fold comprising an antiparallel β-sheet stabilized (β1 and β2) formed by Cys^I^-Cys^IV^. **(A)** In this conformation of VxXXB VxXXB-C(21–50), the β1 and β2 loops connected to their opposing β3 and β4 *via* two parallel disulfide bonds Cys^I^-Cys^IV^ and Cys^II^-Cys^V^ without the crossing of Cys^III^-Cys^VI^ and Cys^II^-Cys^V^. **(B)** In contrast, Cys^III^-Cys^VI^ makes a knot in ICK toxins like huwentoxin-I (PDB 1QK6). **(C)** Multiple sequence alignments of VxXXB-C(21–50) with GeXXA -C(21–50), other conotoxins with granulin fold N_ext_HVc7.2, ϕ-MiXXVIIA, and human granulin A. **(D)** The superimposition of VxXXB VxXXB-C(21–50) overlays with granulin fold proteins including human granulin A (PDB 2JYE) (RMSD 2.23 Å) (i), N_ext_HVc7.2 (PDB 6Q5Z) (RMSD 3.82 Å) (ii), ϕ-MiXXVIIA (PDB 6PPC) (RMSD 2.79 Å) (iii), leech antistasin (PDB 1SKZ) (RMSD 3.51 Å) (iv), protein with EGF-like such as filbrillin (PDB 2IPX) RMSD 3.05 Å) (v) and the C-lobe of the N-terminal domain of Pirh2 (PDB 2K2C) (RMSD 4.24 Å) (vi). The grey ball represents zinc ion, disulfide bonds are in yellow.

**FIGURE 3:**
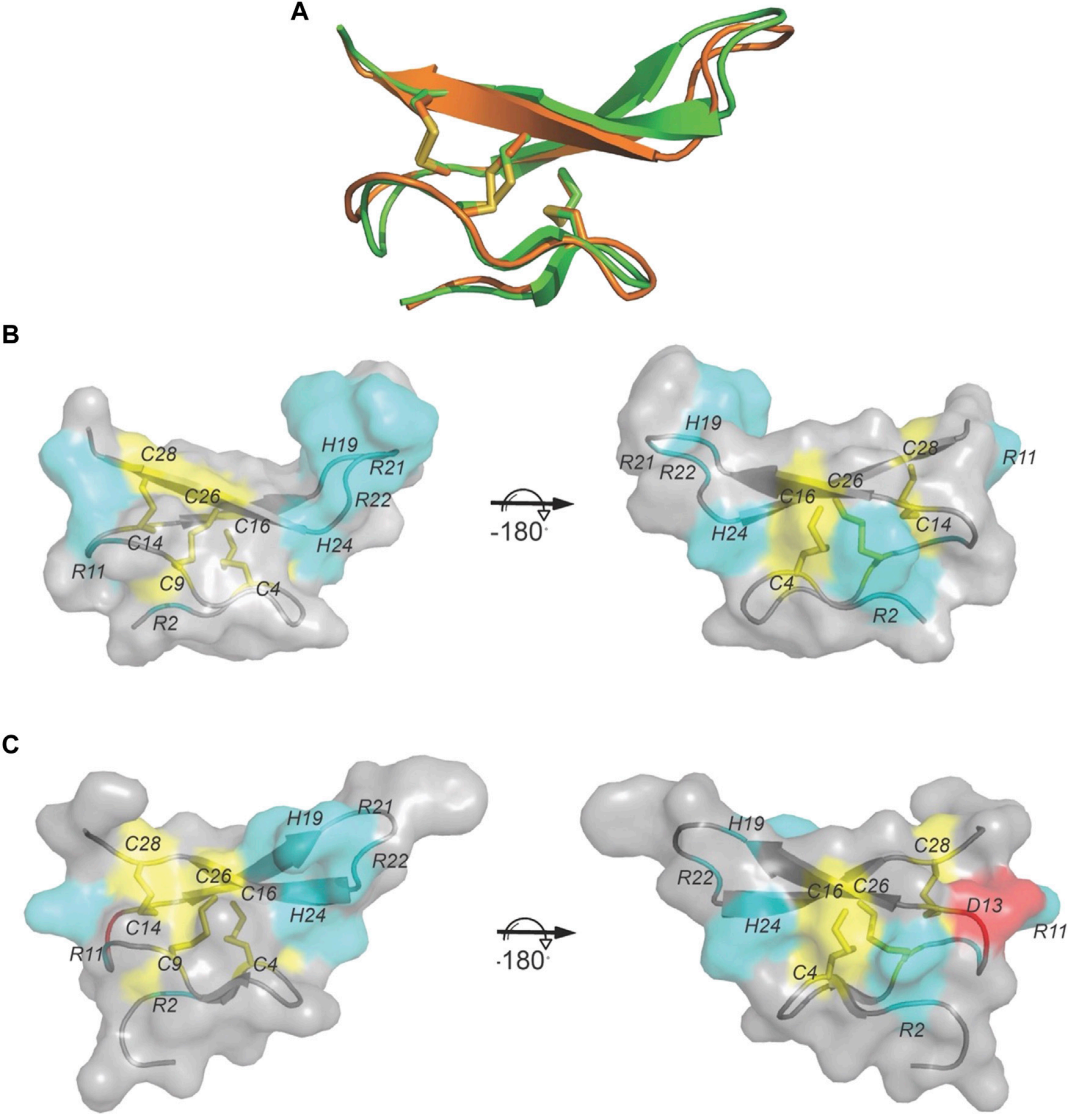
The crystal structure of VxXXB-C (21**–**50). **(A)** VxXXB-C (21**–**50) (orange) shows a high structural similarity to GeXXA-VxXXB-C (21–50) (green) (RMSD 0.56Å). **(B)** Surface of VxXXB-C (21–50) showing loop III contains a positively charged Asp (cyan), while **(C)** the surface of GeXXA-VxXXB-C (21–50) presents a negatively charged Asp in loop III (red) (PDB 4X9Z). Cysteine residues and disulfide bonds are in yellow, positively charged are in cyan and negatively charged are in red.

The sequence of VxXXB-C(21–50) satisfies the ICK peptide consensus sequence C^I^X_3−7_C^II^X_3−6_C^III^X_0−5_C^IV^X_1−4_C^V^X_4−13_C^VI^, where X can be any amino acids VxXXB-C(21–50) is exemplified by double-stranded, antiparallel β-sheets stabilized by the three disulfide bridges (Cys4**–**Cys8, Cys7**–**Cys9, and Cys6**–**Cys10) (Figure 2A). However, the typical secondary structure associated with the ICK fold is not observed in VxXXB-C(21–50). Specifically, β1 and β2 loop are connected to the opposing β3 and β4 *via* the first (Cys^I^ **–**Cys^IV^) and the second disulfide bond (Cys^II^ **–**Cys^V^), respectively. This places the two disulfide bonds in VxXXB-C(21–50) parallel (Figure 2A), in contrast with the crossing pattern of typical ICK peptides (Figure 2B). As a consequence, the third disulfide bond (Cys^III^ **–**Cys^VI^) of VxXXB-C(21–50), which typically threads the loop formed by the first two disulfide bonds to make a knot in the ICK fold (Figure 2B), instead links the β3 loop with the unstructured C-terminal region (Figure 2A). This tertiary structure resembles the N-terminal of the cell proliferation regulator human granulin A (Tolkatchev et al., 2008) and has been previously reported in conotoxins N_ext_H-Vc7.2 and ϕ-MiXXVIIA (Jin et al., 2017; Nielsen et al., 2019). Indeed, with a large second β-hairpin, VxXXB-C(21–50) superimposes the N-terminal of human granulin A (RMSD 2.23 Å), N_ext_H-Vc7.2 (RMSD 3.82 Å) and ϕ-MiXXVIIA (RMSD 2.79 Å). VxXXB-C(21–50) also resembles other granulin-fold proteins including leech antistasins (serine protease inhibitors) (RMSD 3.51 Å) (Lapatto et al., 1997), protein with EGF-like domain such as fibrillin (RMSD 3.05 Å), and the zinc-binding lobe of the human E3 ubiquitin ligase Pirh2 (Sheng et al., 2008), where cysteine residues stabilise zinc ions instead of forming disulfide bonds (RMSD 4.24 Å) (Figure 2D). VxXXB-C(21**–**50) also has high sequence similarity (63%) (Figure 2C) and its bound crystal structure superimposed closely to the C (21–50) domain of GeXXA (RMSD 0.56 Å) (Figure 3A). This similarity suggests αD-conotoxins bind similarly to nAChRs without significant perturbation to the VxXXB-C(21–50) fold, with differences in the number of charged residues in loops II and III expected to influence potency and selectivity (Figures 2C, 3B, C).

#### Structure of CTD (21**–**50) bound to Ls-AChBP

**FIGURE 4:**
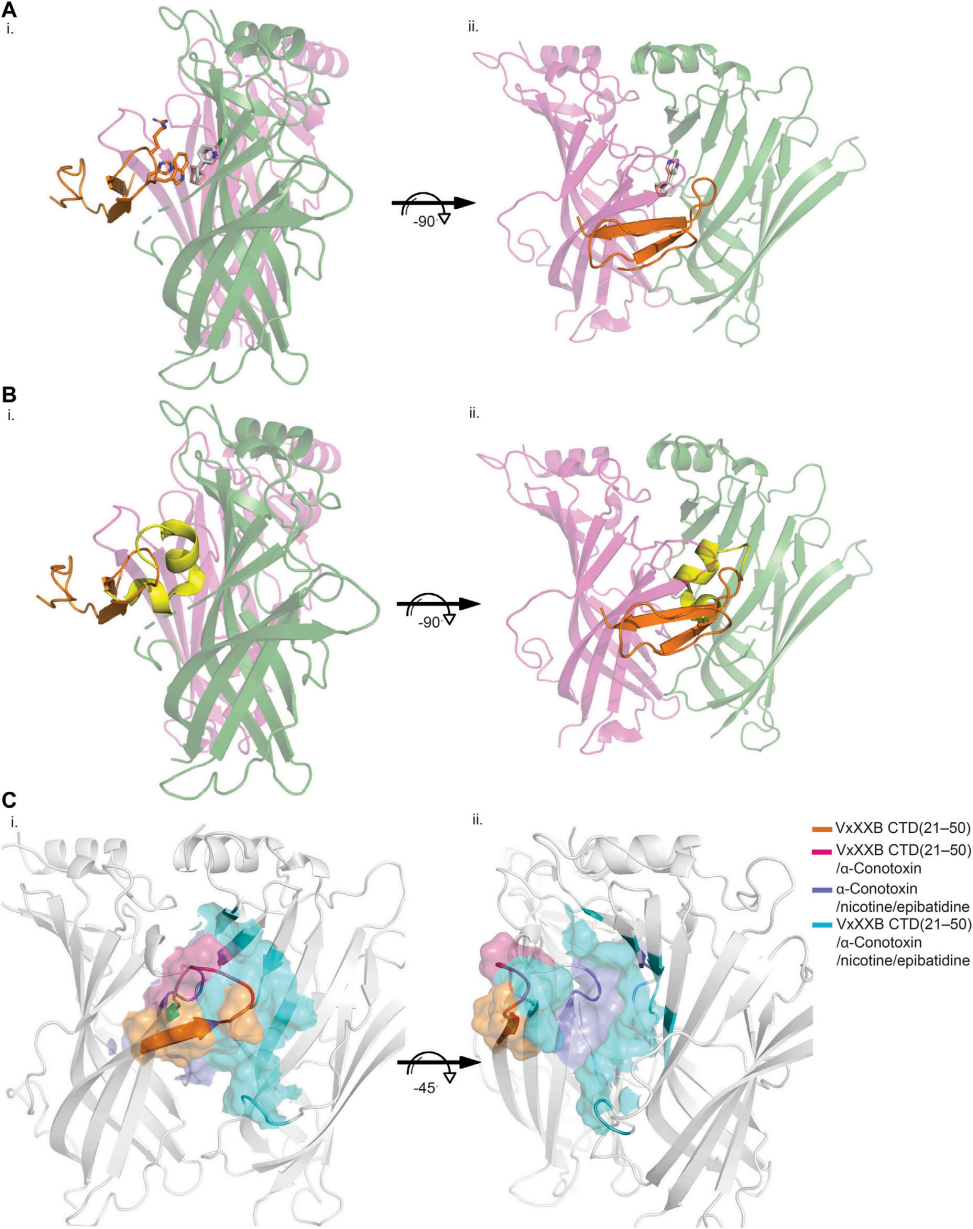
Allosteric binding site of VxXXB-C (21–50) at *Ls*-AChBP. The allosteric binding mode of VxXXB-C (21–50) is revealed through the lack of overlap in binding region with orthosteric agonist nicotine (grey stick) (PDB 1UW6)/epibatidine (pink stick) (PDB 2BYQ) **(A)** and competitive antagonist α-conotoxin LsIA (yellow cartoon) **(B)** viewed from front (i) and side (ii) view as evidenced from the partial overlap in the pairwise interactions between VxXXB-C (21–50) and orthosteric agonist nicotine. **(C)** Interacting regions of VxXXB-C (21–50), α-conotoxins, nicotine are shown with the unique interacting regions of VxXXB-C (21–50) highlighted.

**FIGURE 5:**
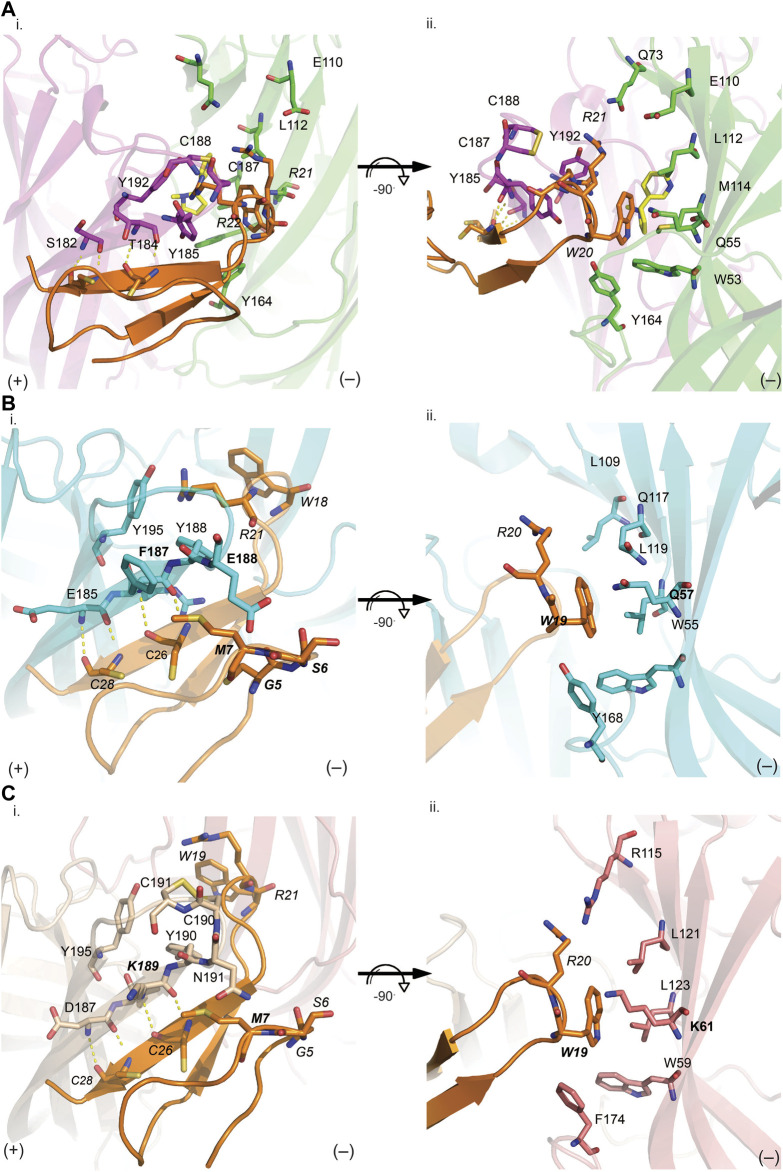
VxXXB-C(21–50) interactions at the principal (i) and complementary (ii) binding site of *Ls*-AChBP **(A)**, human α7 nAChRs **(B)** and human α3β4 nAChRs **(C)**. **(A)** The receptor ligand interactions are uniquely characterized by hydrogen bonds between the β-sheet of CTD (21–50) with the principal binding site (i) and polar bonds on the complementary binding site (ii). The homology models of human α7 receptor **(B)** and human α3β4 nAChRs **(C)** were generated based on the VxXXB-C(21–50)/*Ls*-AChBP co-crystal structure. **(B)** On the principal side (i), α7_F187 and α7_E189 with its long side chains can extend towards VxXXB-C (21–50)_C4, G4, S6, and M7, and exhibit favorable interactions. On the complementary side, similarly, α7_Q57 could interact with VxXXB-C (21–50)_W19. **(C)** Meanwhile, at α3β4 nAChRs complementary face (ii), the corresponding surface is strongly hydrophobic except for the positively charged K61, which may introduce a cation-π interaction with VxXXB-C (21–50)_W19. These interactions likely influence the selectivity of VxXXB-C (21–50) at α7 vs*.* α3β4 nAChRs. VxXXB residues are in italics and major contacts are bolded.

VxXXB-C(21–50) binds to the C-loop of AChBP almost perpendicular to the long axis of the pentamers, making extensive interactions on the principal (+) face of *Ls*-AChBP (Figure 4A). The allosteric binding mode of VxXXB-C(21–50) is confirmed by the lack of overlap of VxXXB-C(21–50) with the orthosteric agonist nicotine (Celie et al., 2004) and epibatidine bound to AChBP (Hansen et al., 2005) (Figures 4A, C). In contrast, CTD (21–50) binding partially overlapped with the orthosteric antagonist α-conotoxin LsIA (Figures 4B, C). VxXXB-C(21–50) binding to the principal face is stabilized through a range of hydrogen bonds and polar interactions, as outlined in Supplementary Table S2. Specifically, the N-terminal β-strand of VxXXB-C(21–50) attaches to *Ls*-AChBP_C-loop *via* four backbone hydrogen bonds between VxXXB-C(21–50)_C28, VxXXB-C(21–50)_C26 and *Ls*-AChBP_S182 and *Ls*-AChBP_T184 respectively, both at 2.9 Å (Figure 5Ai). The insertion of VxXXB-C(21–50) β4 loop into the binding interface allowed VxXXB-C(21–50)_H19 and R22 to approach Y192 and Y185. While these two aromatic residues form the aromatic cage of the orthosteric binding pocket of nAChR that comprises W53, W143, Y192, Y185, the CTD approaches Y192 and Y185 from a different angle to avoid overlap with orthosteric ligands. Other significant interactions at the principal face include cation-π interactions between VxXXB-C(21–50)_R22 and *Ls*-AChBP_Y185 and Y192 (4 Å), and a stacking interaction between VxXXB-C(21–50)_R21 side chain and the vicinal disulfide of C187-C188 of *Ls*-AChBP (3.3 Å) (Figure 5Aii). VxXXB-C (21–50)_M7 extends towards the C-loop, displaying weak hydrophobic interactions with *Ls*-AChBP_T184. At the complementary face, the amine of Q55 contributes a cation-π interaction with VxXXB-C(21–50)_W20 (3.6 Å), E110 potentially forms a salt bridge with VxXXB-C(21–50)_R21 (5 Å) (Figure 5Ai), while the hydroxyl of VxXXB-C (21–50)_Y18 is surrounded by polar S162, E163 and Y164 of *Ls*-AChBP that further stabilize binding (Figure 5Aii).

### Homology model of VxXXB-C(21–50) at α7 and α3β4 nAChRs

VxXXB-C(21–50) exhibited substantial activity at α7 nAChRs but no activity at α3β4 nAChRs. To determine the key determinants in the selectivity of VxXXB-C(21–50) towards α7 nAChRs vs. α3β4 nAChRs, homology models of VxXXB-C(21–50) bound to α7 nAChRs and α3β4 nAChRs were generated using VxXXB-C(21–50)/*Ls*-AChBP co-crystal structure as a template (sequence alignment reported in Supplementary Figure S2). The main hydrogen bonding interactions between VxXXB-C(21–50)_β-strand and nAChR-β-strand remain in two homology models. At the principal binding face, the interacting residues on the α7 nAChR homology model include prominent negatively charged E185, E189, positively charged R186, and hydrophobic F187 and Y188 (Figure 5Bi). In contrast, the equivalent surface on the α3β4 nAChR model comprises negatively charged D187, positively charged K189, polar N191 and hydrophobic I188 and Y190 (Figure 5Ci). At the complementary binding face few major contacts were identified between VxXXB-C(21–50) and human α7 nAChRs, although these surfaces are comparable to the polar (Q57, L109, and Q117) and hydrophobic (W55, L119, and Y168) surfaces of *Ls*-AChBP (Figure 5Bii). At α3β4 nAChRs, the corresponding surface is strongly hydrophobic except for the positively charged K61, which may introduce a cation-π interaction with VxXXB-C(21–50)_W19 (Figure 5Cii). To validate the role of predicted binding determinants, we constructed the α7-like mutants [T184F]*Ls*-AChBP and [S186E]*Ls*-AChBP, and the α3β4-like mutants [T184K]*Ls*-AChBP and [Q55K]*Ls*-AChBP and determined their effect on pharmacology determined, as outlined below.

### Pharmacology of VxXXB-C(21–50) and VxXXB-C(19–50) at *Ls*-AChBP mutants

**FIGURE 6:**
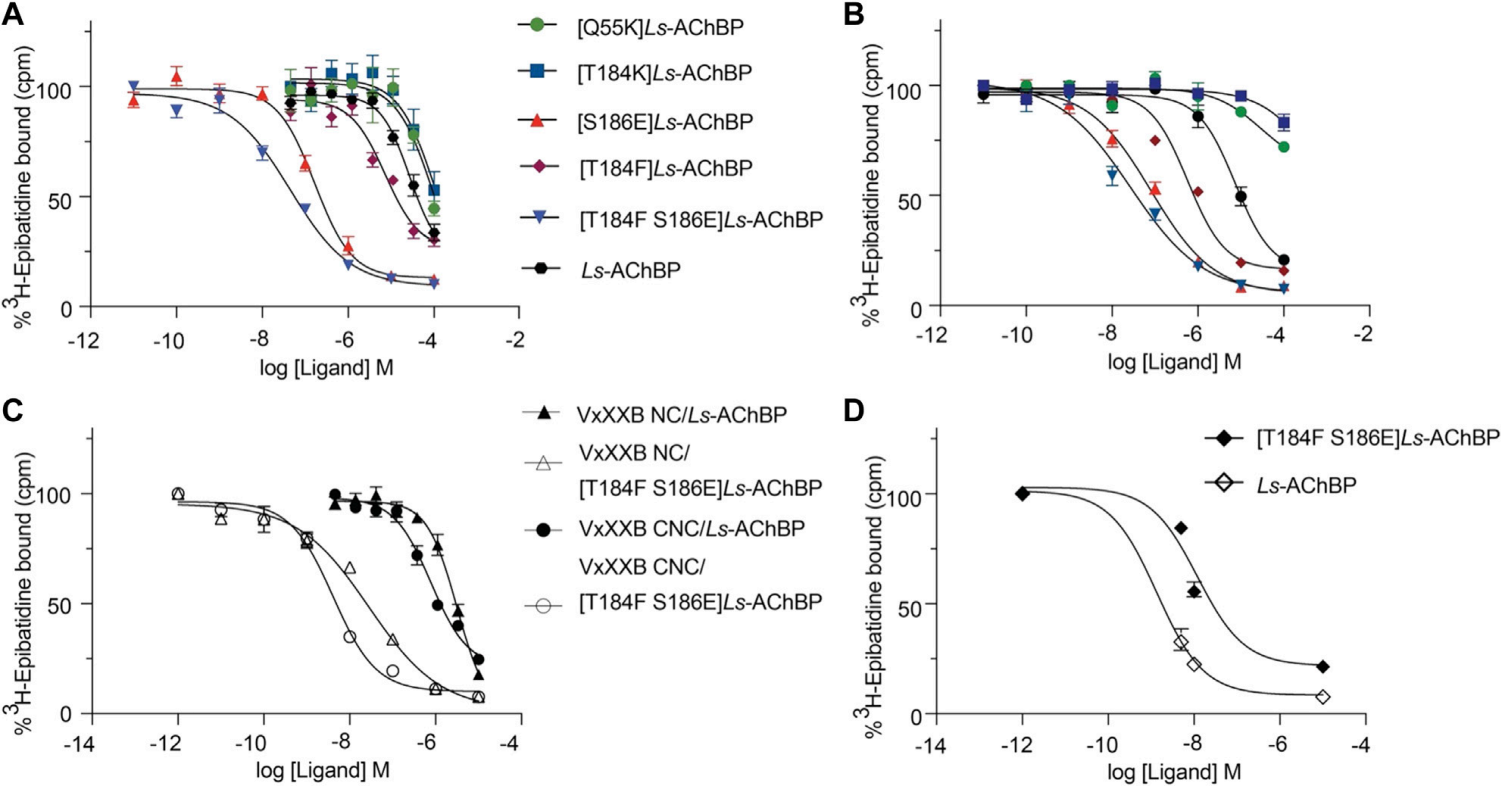
The displacement of [^3^H]-epibatidine from wild-type and mutant *Ls*-AChBP by VxXXB-C (19–50) **(A)**, CTD (21–50) **(B)**, VxXXB-NC **(C)**, VxXXB-CNC **(C)** and native VxXXB **(D)** obtained *via* a competition radioligand binding assay (due to limited sample, top of curve was fixed to 100%, and slope and curve bottom fixed to the bottom of VxXXB CNC**).** Data represent means ± SEM of triplicate data from three independent experiments.

We examined the binding affinity of both CTD variants, including CTD (21–50) and the full-length VxXXB-C(19–50) with four disulfide bonds on mutant *Ls*-AChBPs. The binding affinity of nicotine was unchanged at these mutant *Ls*-AChBP, confirming these positions had no effect on the orthosteric binding site. VxXXB-C(19–50) binding affinity was enhanced 1.3-fold (*p* > 0.05) and 87-fold (*p* < 0.05) at the α7-like mutants [T184F]*Ls*-AChBP and [S186E]*Ls*-AchBP mutants, respectively. In contrast, α3β4-like [T184K]*Ls*-AChBP and [Q55K]*Ls*-AChBP mutants reduced VxXXB-C(21–50) binding affinity by 55-fold and 36-fold (*p* < 0.05), respectively (Figure 6A; Table 1). Remarkably, VxXXB-C(21–50) binding affinity increased 200-fold at α7-like double mutant [T184F S186E]*Ls*-AChBP (*p* < 0.05), supporting the binding pose of VxXXB-C(21–50) in the co-crystal structure and our α7 nAChRs binding pose (Table 1). The binding affinity of full-length VxXXB-C(19–50) at mutant *Ls*-AChBP revealed a similar profile of potency shifts to VxXXB-C(21–50) (Figure 6B; Table 2). VxXXB-C(19–50) showed decreased binding affinity at α3β4-like mutants [Q55K]*Ls*-AChBP and [T184K]*Ls*-AChBP [29.7-fold and >100-fold, respectively (*p* < 0.05)] and enhanced binding affinity for α7-like mutants [S186E]*Ls*-AChBP, [T184F]*Ls*-AChBP and [T184F S186E]*Ls*-AChBP (80-, 12.4- and 240-fold, respectively) compared to wildtype *Ls*-AChBP (*p* < 0.05).

**TABLE 1:** IC_50_ values for displacement of [^3^H]-epibatidine binding to *Ls*-AChBPs and mutant *Ls*-AChBPs by CTD (19–50), VxXXB-C (21–50), VxXXB-NC and VxXXB-CNC.

	CTD (21–50)		VxXXB-C (19–50)		VxXXB-NC		VxXXB-CNC		Native VxXXB	
	IC_50_ ± SEM (µM)	Ratio*	IC_50_ ± SEM (µM)	Ratio*	IC_50_ ± SEM (µM)	Ratio*	IC_50_ ± SEM (µM)	Ratio*	IC_50_ ± SEM (µM)	Ratio*
*Ls*-AChBP	10 ± 0.23	1	7.2 ± 0.87	1	2.3 ± 0.4	1	0.8 ± 0.09	1	0.011	1
[Q55K]*Ls*-AChBP	36.0 ± 0.98	36**	29.7 ± 2.12	29.7**						
[S186E]*Ls*-AChBP	0.04 ± 0.005	0.004**	0.09 ± 0.001	0.01**						
[T184K]*Ls*-AChBP	55.0 ± 10.2	55**	>100	>100**						
[T184F]*Ls*-AChBP	7.2 ± 0.872	1.38	0.58 ± 0.02	0.08**						
[T184F S186E]*Ls*-AChBP	0.05 ± 0.002	0.005**	0.03 ± 0.004	0.004**	0.03 ± 0.008	0.01**	0.004 ± 0.0005	0.005**	0.001	0.09

**Indicates significant difference in IC_50_ values to wildtype *Ls*-AChBP, at (*p* < 0.05).

**TABLE 2:** IC_50_ values for displacement of [^3^H]-epibatidine binding to *Ls*-AChBPs and mutant *Ls*-AChBPs by CTD (19–50), VxXXB-NC and VxXXB-CNC.

	VxXXB-C(19–50)		VxXXB-NC		VxXXB-CNC	
	IC50± SEM (µM)	Ratio*	IC_50_ ± SEM (µM)	Ratio*	IC50± SEM (µM)	Ratio*
*Ls*-AChBP	10 ± 0.23	1	10.0 ± 2.00	1	0.8 ± 0.09	1
[H69A]*Ls*-AChBP	10 ± 0.23	1	10.0 ± 2.00	1	>10	>10**
[R23D]*Ls*-AChBP	10 ± 0.23	1	10.0 ± 0.90	1	>10	>10**

**Indicates significant difference in IC_50_ values to wildtype *Ls*-AChBP, at (*p* < 0.05).

To confirm the binding determinants for the CTD domain extended to larger and full-length αD-conotoxin constructs, the binding affinity of VxXXB-NC and full-length VxXXB-CNC were determined at the α7-like [T184F S186E]*Ls*-AChBP. As observed for the CTD domain, VxXXB-NC and VxXXB-CNC also showed an increased affinity at this mutant (76.7–fold and 200-fold, respectively) (Figure 6C; Table 1). Similarly, the estimated potency of native VxXXB also increased ∼10-fold at α7-like [T184F S186E]*Ls*-AChBP compared to wild-type *Ls*-AChBP (Figure 6D).

### Proposed binding mode of the full-length native homodimeric αD-VxXXB

**FIGURE 7:**
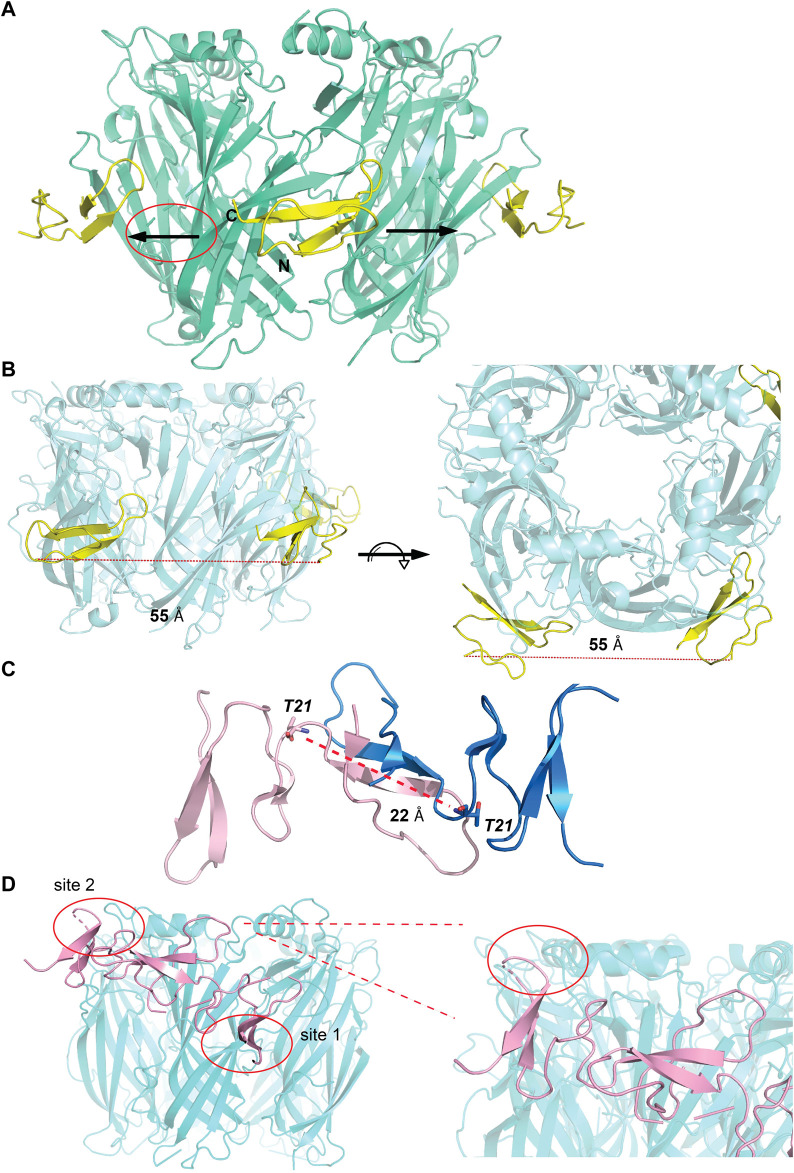
Potential binding mode of homodimeric VxXXB. **(A)** A clockwise orientation was proposed for VxXXB if occupying two adjacent binding sites. **(B)** Side and top view of the *Ls*-AChBP/αD-VxXXB-C (21–50) co-crystal structure. The distance between two adjacent αD-VxXXB-CTDs is 55 Å assuming a linear approach. **(C)** The distance between two ends of αD-VxXXB-CTD (as measured between Thr21 from each end) is 22 Å. **(D)** The docking result of homodimeric VxXXB model built from the crystal structure of GeXXA at *Ls*-AChBP is displayed. Docking was performed using the HADDOCK webserver and residues found in the interactions between αD-VxXXB-C (21–50) and *Ls*-AChBP were used as constraints. The second CTD interacting with binding site 2 is circled and *Ls*-AchBP_Asp5, equivalent to rat α10_His7 that interacted with GeXXA, is shown in stick view.

The observations from the VxXXB-C(21–50)/*Ls*-AChBP co-crystal structure, together with functional data previously reported (Ho et al., 2022), were used to formulate the potential binding mode of native homodimeric VxXXB (Table 2). The comparable potency of VxXXB-C(19–50) and VxXXB-NC, and the 2-fold enhance potency of VxXXB-CNC suggests that NTD mainly acts to facilitate cooperative binding between the two CTDs. The VxXXB-C(21–50) binding orientation observed in the co-crystal structure allows full-length VxXXB to extend in a clockwise direction towards a second distinct CTD binding site (Figure 7A). As the distance between the two C-loop binding sites is ∼55 Å (Figures 7A, B), VxXXB-C (∼22 Å) (Figure 7C) cannot span these on homomeric *Ls*-AChBP or α7 nAChRs. Given these considerations, we propose that one CTD binds to the C-loop (site 1), while the second CTD binds to the adjacent binding interface at a different site (site 2) (Figure 7D).

**FIGURE 8:**
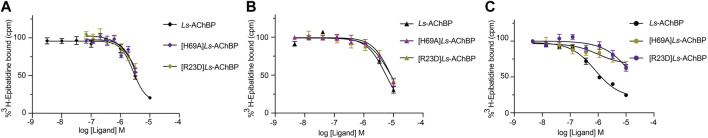
Concentration-response curves of VxXXB-C (19–50) **(A)**, VxXXB-NC **(B)** and VxXXB-CNC **(C)** at wild-type and mutant *Ls*-AChBP, [H69A]*Ls*-AChBP and [R23D]*Ls*-AChBP obtained *via* a competition radioligand binding assay.

To further evaluate the binding of full length αD-conotoxins, we docked modelled VxXXB at human α7 nAChRs. Using mutational data obtained from the VxXXB-C(21–50)/*Ls*_AChBP complex as constraints, our docking results generated a pose where one VxXXB CTD binds at the outer face of the C-loop that overlapped but was distinct from the CTD orientation observed in the co-crystal structure. This pose allowed the second CTD of VxXXB to extend up and across towards the N-terminal region of loop D of the adjacent α7 subunit in a clockwise direction from site 1 (Figure 7D). Unfortunately, due to insolubility issues we were unable to generate useful co-crystals of VxXXB-NC or VxXXB-CNC with AChBP that might provide further resolution of these differences. However, these docking support mutations identified near the N-terminal end of the adjacent subunit that affect GeXXA potency (Xu et al., 2015; Yang et al., 2017). To further confirm the location of binding site 2, we mutated two residues on top of nAChRs predicted to affect the binding of full-length VxXXB but not VxXXB-NC and VxXXB-C (19–50) binding, [H69A]*Ls*-AChBP and [R23D]*Ls*-AChBP (Supplementary Figure S2). As predicted, both the [H69A]*Ls*-AChBP mutation on the complementary side and the [R23D]*Ls*-AChBP mutation on the principal side reduced affinity for VxXXB-CNC, while the affinities of VxXXB-C(19–50) and VxXXB-NC remained unchanged (Figure 8; Table 2).

## Discussion

Many conotoxin families have been identified to target nAChRs, including the extensively studied α-conotoxins, which are competitive inhibitors acting at the orthosteric site (Lebbe et al., 2014). The ψ-, αB-, αC-, αD-, and αS-conotoxins are also functional antagonists but their binding sites on nAChRs are currently unknown (Shon et al., 1997; Jimenez et al., 2007; Luo et al., 2013; Christensen et al., 2015). As AChBPs are homologous to the extracellular ligand-binding domain of the nAChR, especially α7 nAChR, these proteins provide useful structural templates for modeling the ligand-binding domain of mammalian nAChRs. Thus, in this study, we define the allosteric binding site for αD-conotoxins from the co-crystal structure of granulin domain VxXXB-C(21–50) bound to *Ls*-AChBP.

In the co-crystal complex of VxXXB-C(21–50)/*Ls*-AChBP, despite possessing the ICK cysteine connectivity, VxXXB-C(21–50) adopts a granunlin-like structure comprising an antiparallel β-sheets stabilized by cystine bonds (Figure 2) (Moore et al., 2012). Granulins are ancestral ∼55-residue growth factors responsible for development and wound healing. The N-terminal ∼30 residues of granulins includes the characteristic core granulin fold identified previously in conotoxins Φ-MiXXVIIA and N_ext_H-Vc7.2 as a mini-granulin fold distinct from the ICK toxin fold (Tolkatchev et al., 2008; Palfree et al., 2015; Jin et al., 2017; Nielsen et al., 2019). Indeed, mini-granulin-fold proteins (Sheng et al., 2008) were used by AlphaFold to model VxXXB from human and *Drosophila* E3 ubiquitin-protein ligase protein (Ho et al., 2022). Unlike Φ-MiXXVIIA and N_ext_H-Vc7.2, which have few residues in loop III and more residues in loop IV, VxXXB-C(21–50) has most residues in its negatively charged loop III, which allows closer alignment to the core motif of granulin A. Importantly, loop III of VxXXB-C contributed most interactions at site 1 and thus appears mainly responsible for VxXXB-C(21–50) affinity (Figures 3B, 5). Indeed, proposed functional determinants in granulins (R15, L16, and S17 in granulin A) (Palfree et al., 2015) align with the key binding determinants (Y18, H19, and R21) in VxXXB-C (Figure 2C), suggesting repurposing of this region of loop III to target nAChRs. Superimposition of the crystal structures of GeXXA, VxXXB-C(21–50) and GeXXA-C(21–50) revealed high structural similarity (Figure 3A), which is little altered upon binding to loop C. However, differences in loop III residues may underlie the lower potency of GeXXA at human α7 nAChRs compared to VxXXB [210 nM (Xu et al., 2015) vs*.* 0.4 nM (Loughnan et al., 2006)].

As observed from the co-crystal structure, VxXXB-C(21–50) anchors on the outer edge of C-loop *Ls*-AChBP *via* four hydrogen bonds between its β-strand backbone and β-strand of *Ls*-AChBP_C-loop and β4-loop inserting into the binding interface. An outward displacement of *Ls*-AChBP_C-loop, which is characteristic of antagonist binding in the orthosteric site, is also displayed by VxXXB-C(21–50), suggesting allosteric antagonists can also inhibit nAChRs by stabilizing the open (extended) C-loop. At the principal binding site, the β4-loop of VxXXB-C(21–50) contacts the binding interface, allowing VxXXB-C(21–50) to interact perpendicular to the long axis of AChBP with only two (Y185 and Y192) (Figure 5Ai) of the four (W53, Y185, Y192, and W143) aromatic residues forming the orthosteric binding site, and thus avoid overlapping with the binding of orthosteric ligands both agonists (Figure 4A) and antagonists (Figure 4B) that typically position deep within this aromatic cage. This observation is in line with the IUP definition of non-competitive/allosteric antagonism, where agonist and antagonist can be bound to the receptor simultaneously and antagonist binding reduces or prevents the action of the agonist with or without any effect on the binding of the agonist (Neubig et al., 2003), confirming the allosteric binding mode of the CTD of αD-conotoxins (Figure 4C). Interestingly, VxXXB-C (21–50) stacks against the vicinal disulfide bond of *Ls*-AChBP_C-loop *via* R21 (Figure 5Aii) instead of the typical first disulfide bond of α-conotoxins, reinforcing the importance of C-loop vicinal disulfide bond targeting by conotoxins at nAChRs A number of interactions between VxXXB-C(21–50) and the complementary binding face (Q55, Q73, E110, L112, M114, S162, and Y164) also overlap with classical α-conotoxins (Figure 5A and Supplementary Table S2), consistent with β2_L109Q mutation (equivalent to *Ls*-AChBP_L112) slowing recovery of α3β2 nAChRs from native VxXXB block (Loughnan et al., 2006).

Homology models of VxXXB-C(21–50) bound at human α7 and α3β4 nAChRs were built using the co-crystal complex VxXXB-C(21–50)/*Ls*-AChBP as a template to reveal the key determinants of VxXXB-C(21–50) potency at human α7 vs*.* α3β4 nAChRs. The unique backbone hydrogen bonding interactions between VxXXB-C(21–50)_β-strand and C-loop_β-strand remain in both models. Important interactions observed at α7 and α3β4 nAChR models were confirmed *via* functional assays on α7-like and α3β4-like *Ls*-AChBP mutations. The most significant effects were observed for the double-mutant α7-like [T184F S186E]*Ls*-AChBP, where the mutated residues are located on the outer face of loop C (Figure 6; Table 1). Meanwhile, the binding affinity of VxXXB-C(21–50) decreased at [Q55K]*Ls*-AChBP, suggesting this interaction instead of forming a cation-π interaction may introduce an electrostatic clash that is responsible for the poor binding of VxXXB-C(21–50) at α3β4 nAChRs. Importantly, T184F and S186E also affected the binding affinity of both VxXXB-CNC and native VxXXB. From these data, we propose that full-length VxXXB interacts with nAChRs at two distinct sites that facilitates cooperative binding between the two CTDs. Cooperative binding has been previously observed for dendrimers of α-conotoxins ImI, Vc1.1, RgIA, and PeIA coupled through an ∼ 32 Å linker that allowed simultaneous binding across adjacent orthosteric sites on homomeric nAChRs ^18,19^. However, the shorter equivalent linker in VxXXB (22 Å between dendrimers) cannot span two adjacent orthosteric sites. It appears that the first CTD is the main component responsible for the potency of full-length VxXXB, while the second CTD provides additional interactions facilitated by the NTD linker. This was supported by the rank order of potency of the different constructs, with full-length synthetic VxXXB CN(1–18)C > VxXXB-N(1–18)C > VxXXB-C(19–50), while full-length synthetic VxXXB CN(1–18)C was only 12-fold more potent than VxXXB-N (1–18)C (Table 2). As such, full-length VxXXB would occupy two binding sites simultaneously, stabilising at nAChR with one CTD of VxXXB initially binding to the C-loop of the principal binding face (site 1), followed by the second CTD extending to contact the adjacent subunit binding interface in a clockwise direction (extracellular view). Indeed, the four hydrogen bonds identified between β-sheet of VxXXB-C and the C-loop of nAChRs, as well as the interactions VxXXB-C with α7_F187 and E189, are the key determinants that are likely account for the stronger interactions between VxXXB and site 1 compared to site 2. A complex of *Ls*-AChBP with VxXXB-C (21–50) occupying the second binding site was not detected during our co-crystallisation attempts, supporting a secondary role for site 2 to enhance VxXXB potency. We propose that VxXXB binding to site 1 trap the C-loop of nAChRs in an extended (open) state that has low orthosteric agonist and antagonist affinity, while binding at site 2 likely only influence VxXXB affinity. Further studies on the binding kinetics of VxXXB-C(21–50) as well as its influence on transitions between operating states are expected to shed further light on the allosteric mechanism of action of αD-conotoxins.

The docking results of the modelled full-length VxXXB at human α7 nAChRs support this two-site binding hypothesis, with one CTD binding to the outer face of C-loop as seen in the co-crystal complex (site 1), and the second CTD contacting the N-terminal region of loop D of the adjacent α7 subunit clockwise from site 1 (site 2), resulting in distinct sets of pairwise interactions at the two binding sites. Consistent with this conclusion, mutations at the top of the α9α10 nAChR were found to affect GeXXA potency (Xu et al., 2015). Our proposed binding mode was further supported by the loss in binding affinity by full-length VxXXB-CNC at [H69A]*Ls*-AChBP and [R23D]*Ls*-AChBP at the top of the receptor, while the affinity of the shorter VxXXB-NC and VxXXB remained unchanged. Our data are inconsistent with the earlier proposal that GeXXA forms a lid-covering covering the entrance to the ion conducting pore (Yang et al., 2017). Interestingly, the nAChR residues interacting with VxXXB at both CTD binding sites vary across neuronal nAChR subtypes. In this case, α7-specific interactions are the possible hydrophobic interaction between α7_F187 and VxXXB-C(21–50)_M7 and G5, and polar interaction between α7_E189 long side chain and VxXXB-C (21–50)_S6, which can offer opportunities for the rational design of novel allosteric inhibitors with shifted subtype selectivity (Supplementary Figures S2, S3). Importantly, these allosteric binding sites are highly conserved among neuronal α7 nAChR subunit of different species, suggesting VxXXB could inhibit α7-like nAChR subtypes of different species, including worms that are hunted by *C. vexillum*.

Previously, we found that VxXXB-C (19–50) was only 2-fold less potent that full-length VxXXB-CNC (Figure 1A) at allosterically inhibiting α7 nAChRs, indicating that a single CTD is sufficient for full function. Given the reduced synthetic challenges associated with constructing the CTD vs. full-length VxXXB, the granulin-like CTD presents an attractive starting point for the design of novel allosteric antagonists at nAChRs. The binding mode of VxXXB-C (21–50) and its pair-wise interactions with AChBP could guide the rational design of VxXXB-C (21–50) and VxXXB-C (19–50) analogues to further enhance their potency, specificity and therapeutic potential to modify nAChR-related diseases.

## Conclusion

In this study we define the binding mode of the CTD of αD-VxXXB- from its co-crystal structure with *Ls*-AChBP and use mutational studies to confirm the binding mode of full length VxXXB-CNC. Our findings reveal that the CTDs of native homodimeric αD-VxXXB bind cooperatively at two novel allosteric sites on nAChRs, one on the outer edge of C-loop and the second clockwise and up towards the N-terminal of loop D of the adjacent subunit. The CTD of VxXXB adopts a granulin fold stabilized by a third disulfide bond that offers new avenues for stabilizing granulin-like peptides and provides a new avenue for the design of new sub-type selective allosteric nAChR antagonists.

## Data Availability

The datasets presented in this study can be found in online repositories. The names of the repository/repositories and accession number(s) can be found below: RSCB PBD [https://www.rcsb.org/], 7TXF.

## Abbreviations

AChBP, acetylcholine binding protein; Acm, S-acetamidomethyl; ACN, acetonitrile; CD, circular diochroism; IMAC, immobilized metal affinity chromatography purification; *Ls, Lymnaea Stagnalis*; MeOH, methanol; nAChRs, nicotinic acetylcholine receptors; PNU120596, N-(5-Chloro-2,4-dimethoxyphenyl)-N′-(5-methyl-3-isoxazolyl)-urea; TFA, trifluoroacetic acid; Trt, trityl; Ub, Ubiquitin.
