# Supplementary material for: Unravelling the allosteric binding mode of αD-VxXXB at nicotinic acetylcholine receptors
Source: Front Pharmacol. 2023 Apr 13;14:1170514. doi: 10.3389/fphar.2023.1170514 (PMC10133702; doi:10.3389/fphar.2023.1170514)
Supplement: Supplementary file 1 [file DataSheet1.PDF]

Supplementary data

Unravelling the allosteric binding mode of αD-conotoxin VxXXB at nicotinic acetylcholine receptors

Thao NT Ho, Nikita Abraham, Richard J. Lewis*

Centre for Pain Research, Institute for Molecular Bioscience, The University of Queensland, St Lucia, QLD, Australia

***Correspondence:**
Richard J. Lewis

r.lewis@uq.edu.au

Keywords: α-conotoxin, α7 nAChR, pharmacology, AChBP, allosteric

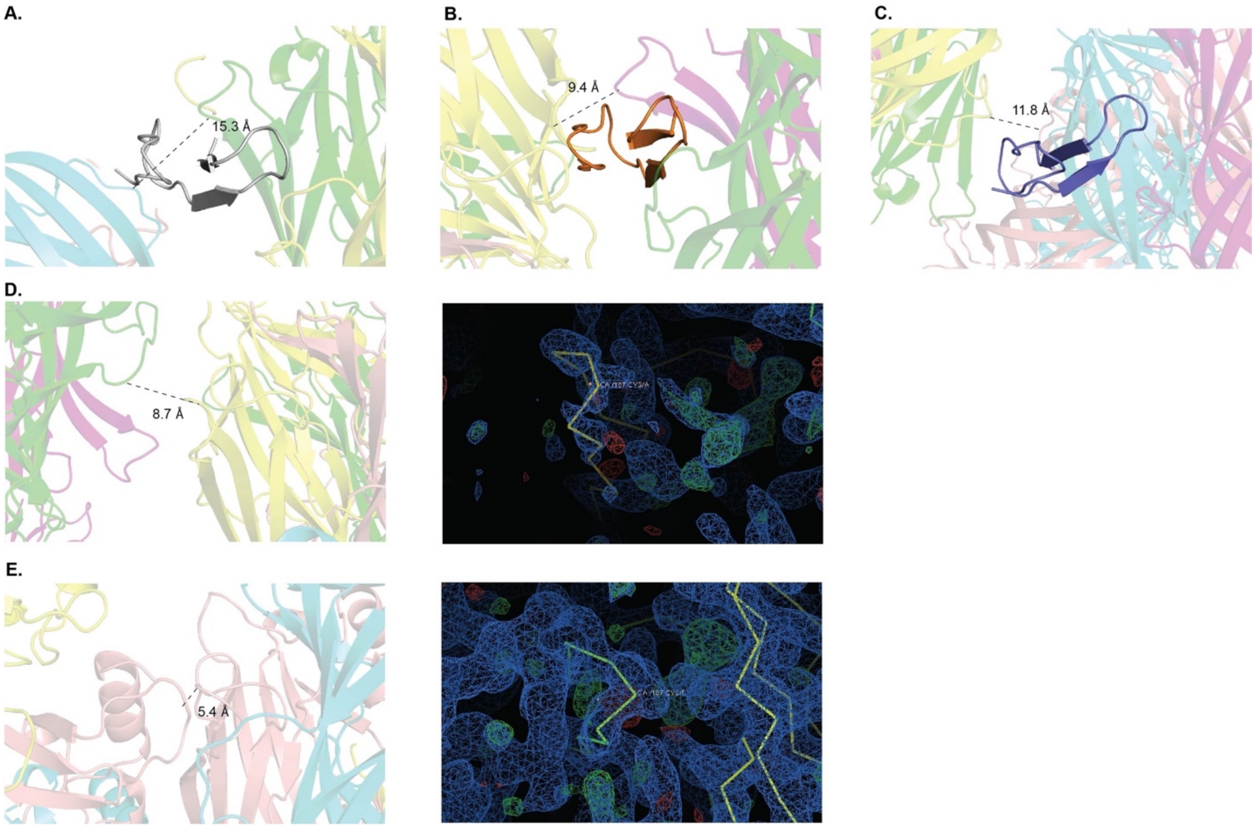

Fig. S1. The effects of crystal packing on VxXXB CTD(21–50) at the binding interface of *Ls*-AChBP. More space is seen between the ligand from the pentamer and the adjacent crystal mate, consistent with the defined electron density for the ligand only being observed at this binding pocket (A, B, C). Meanwhile, the other binding interfaces are either with reduced ligand densities (D) or partially hindered by the adjacent crystal mate (E), consistent with the weaker electron density in these binding pockets (15.3 Å, 9.4 Å and 11.8 Å from occupied sites *vs*. 5.3 Å for the unoccupied site and 8.7 Å for the partially occupied site, measured from Ser186 of occupied binding interface and the closest residue on the C-loop of the adjacent crystal mate).

```
                                                                          D
Ls-AChBP        ---LDR---ADILYNIRQTSRPDVIPTQRDRPVAVSVSLKFINILEVNEITNEVDVVFWQ   55
α3              --SEAEHRLFERLF--EDYNEIIRPVANVSDPVIIHFEVSMSQLVKVDEVNQIMETNLWL   56
β2              --TDTEERLVEHLLDPSRYNKLIRPATNGSELVTVQLMVSLAQLISVHEREQIMTTNVWL   58
β4              RVANAEEKLMDDLLNKTRYNNLIRPATSSSQLISIKLQLSLAQLISVNEREQIMTTNVWL   60
α7              --GEFQRKLYKELV--KNYNPLERPVANDSQPLTVYFSLSLLQIMDVDEKNQVLTTNIWL   56
α9              ADGKYAQKLFNDLF--EDYSNALRPVEDTDKVLNVTLQITLSQIKDMDERNQILTAYLWI   58
α10             AEGRLALKLFRDLF--ANYTSALRPVADTDQTLNVTLEVTLSQIIDMDERNQVLTLYLWI   58
                            *      .       . .  : : . :.: :: .:.*  : :   .*

                                           A             E
Ls-AChBP        QTTWSDRTLAWNSSHSP--DQVSVPISSLWVPDLAAYNAISK-PEVLTPQLARVVSDGEV  111
α3              KQIWNDYKLKWNPSDYGGAEFMRVPAQKIWKPDIVLYNNAVGDFQVDDKTKALLKYTGEV  116
β2              TQEWEDYRLTWKPEEFDNMKKVRLPSKHIWLPDVVLYNNADGMYEVSFYSNAVVSYDGSI  118
β4              KQEWTDYRLTWNSSRYEGVNILRIPAKRIWLPDIVLYNNADGTYEVSVYTNLIVRSNGSV  120
α7              QMSWTDHYLQWNVSEYPGVKTVRFPDGQIWKPDILLYNSADERFDATFHTNVLVNSSGHC  116
α9              RQIWHDAYLTWDRDQYDGLDSIRIPSDLVWRPDIVLYNKADDESSEPVNTNVVLRYDGLI  118
α10             RQEWTDAYLRWDPNAYGGLDAIRIPSSLVWRPDIVLYNKADAQPPGSASTNVVLRHDGAV  118
                   * *  * *. .     . : .*   :* **:  **               :   *

                                              B          β8         F
                                                     ___________
Ls-AChBP        LYMPSIRQRFSCDVSGVD-TESGATCRIKIGSWTHHSREISVDPTTENSDDSEYFSQYSR  170
α3              TWIPPAIFKSSCKIDVTYFPFDYQNCTMKFGSWSYDKAKIDLVLIGSSMNLKD-YWESGE  175
β2              FWLPPAIYKSACKIEVKHFPFDQQNCTMKFRSWTYDRTEIDLVLKSEVASLDD-FTPSGE  177
β4              LWLPPAIYKSACKIEVKYFPFDQQNCTLKFRSWTYDHTEIDMVLMTPTASMDD-FTPSGE  179
α7              QYLPPGIFKSSCYIDVRWFPFDVQHCKLKFGSWSYGGWSLDLQM--QEADISG-YIPNGE  173
α9              TWDAPAITKSSCVVDVTYFPFDNQQCNLTFGSWTYNGNQVDIFNALDSGDLSD-FIEDVE  177
α10             RWDAPAITRSSCRVDVAAFPFDAQHCGLTFGSWTHGGHQLDVRPRGAASLAD-FVENVE  177
                 :      : :* :.      .   * :.: **::   .:.:       .    :    .

                       β9          C      β10
                _____________   __________________
Ls-AChBP        FEILDVTQKKNSVTYSCCPEAYEDVEVSLNFRKKGRSEIL          210
α3              WAIIKAPGYKHDIKYNCCEEIYPDITYSLYIRRL------          209
β2              WDIVALPGRRNE---NPDDSTYVDITYDFIIRRK------          208
β4              WDIVALPGRRTV---NPQDPSYVDVTYDFIIKRKPLFYT-          215
α7              WDLVGIPGKRSERFYECCKEPYPDVTFTVTMRRRT-----          208
α9              WEVHGMPAVKNVISYGCCSEPYPDVTFTLLLKRRS-----          212
α10             WRVLGMPARRRVLTYGCCSEPYPDVTFTLLLRRRAA----          213
                : :      :           * *:   . :::
```

Fig. S2. Sequence alignment of *Ls*-AChBP and human neuronal nAChR subunits. Loops A-F, contributing to the binding site, are highlighted in green blocks for princiapl binding site and yellow blocks for complementary binding site. Unique residues interacting with VxXXB are bolded.

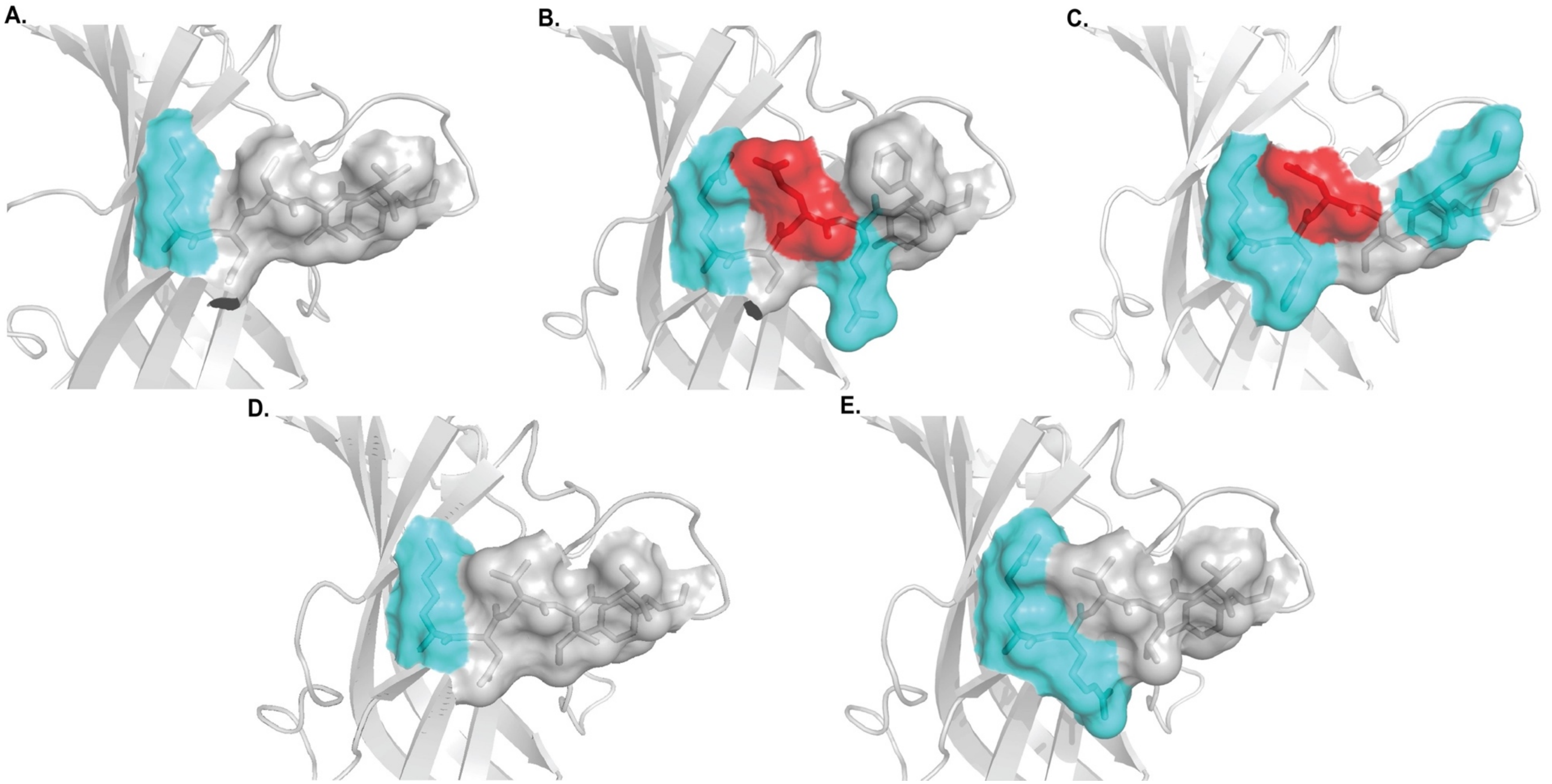

Fig. S3. The surface of allosteric binding site (site 1) of VxXXB CTD at *Ls*-AChBP (A), human α7 nAChRs (B), human α3 nAChRs (C), human α9 nAChRs (D) and human α10 nAChRs (E). Positively charged residues are in cyan and negatively charged residues are in red.

Table S1. Data collection and refinement statistic

	***Ls*-AChBP- VxXXB CTD(21-50)**
**Data collection**	
Space group	$P_{21\ 21\ 21}$
Cell dimensions, Å	a= 68.91 Å, b= 119.57 Å, c= 150.73 Å
Cell dimensions, °	α=90°, β=90°, γ=90°
Resolution, Å	46.84-2.47 (2.55-2.47)
Rsym	0.115 (1.153)
I/Iσ	11.5 (0.9)
Completeness (%)	99.4 (94.6)
Multiplicity	6.7 (6.6)
Total no. of reflections	302408 (27451)
Unique reflections	45467 (4181)
**Refinement**	
Resolution Å	46.84-2.47
Rwork/Rfree	0.1983/ 0.2463
rmsd bond distance, Å	0.015
rmsd bond distance, Å	1.635
Average B-factor	76.7

Table S2. Contacts between *Ls*-AChBP and aD-conotoxins

Ls-AChBP	Nicotine	Epibatidine	α-conotoxins	αD-VxXXB CTD	Distance (Å)
Principal side					
Tyr89	×	×	×		
Ser142	×	×	×		
Trp143	×	×	×		
Thr144	×	×	×		
Lys180				Pro29	2.8 ± 0.03
				Gly30	3.8 ± 0.09
Asn181				Pro29	2.0 ± 0.10
Ser182				Gly30	3.8 ± 0.03
				Cys28	3.1 ± 0.16
				Ser27	3.2 ± 0.21
				Cys26	3.4 ± 0.05
Val183				Val17	4.7 ± 0.21
				Cys26	4.4 ± 0.12
Thr184				Met7	3.6 ± 0.14
				Gly25	2.8 ± 0.03
				Cys26	1.9 ± 0.06
				Ser27	3.2 ± 0.14
Tyr185	×	×	×	Val17	3.4 ± 0.12
				His19	3.2 ± 0.09
				Arg22	3.3 ± 0.76
				Gly25	3.1 ± 0.92
Ser186			×	Met7	3.4 ± 0.53
Cys187	×	×	×	Arg22	3.3 ± 0.13
Cys188	×	×	×	×	
Glu190			×	×	
Tyr192	×		×	Arg22	4.0 ± 0.39
Complementary face					
Trp53	×	×	×		
Gln55				Trp20	3.6 ± 0.64
Gln73			×	Arg21	4.8 ± 0.48
Arg104	×	×	×		
Val106	×	×	×		
Glu110			×	Arg21	5.0 ± 0.12
Leu112	×	×	×	Trp20	3.4 ± 0.28
				Arg21	5.0 ± 0.12
Met114	×	×	×	His19	5.0 ± 0.29
				Trp20	5.0 ± 0.12
Glu163			×		
Tyr164	×	×	×	Tyr18	2.9 ± 0.35
				His19	3.4 ± 0.78
				Trp20	4.1 ± 0.12
